# Supplementary material for: Prevalence of oral submucous fibrosis across diverse populations: a systematic review and meta-analysis
Source: PeerJ. 2024 Nov 6;12:e18385. doi: 10.7717/peerj.18385 (PMC11549909; doi:10.7717/peerj.18385)
Supplement: Supplemental Information 1 [file peerj-12-18385-s001.docx]

**MEDLINE via pubmed 1954**

Searches:

(((((Fibroses, Oral Submucous) OR (Fibrosis, Oral Submucous)) OR (Oral Submucous Fibroses)) OR (Submucous Fibroses, Oral)) OR (Submucous Fibrosis, Oral)) OR ("Oral Submucous Fibrosis"[Mesh])

Date Run: Sun Mar 31 2024 20:33

**Results: 1954**

**Database: Web of Science Core Collection**

Searches:

(((((ALL=(Oral Submucous Fibrosis)) OR ALL=(Fibroses, Oral Submucous)) OR ALL=(Fibrosis, Oral Submucous)) OR ALL=( Oral Submucous Fibroses)) OR ALL=( Submucous Fibroses, Oral)) OR ALL=(Submucous Fibrosis, Oral)

Date Run: Sun Mar 31 2024 20:10

**Results: 1731**

**Database: Cochrane Central Register of Controlled Trials (CENTRAL)**

Searches:

#1 (Submucous Fibrosis, Oral):ti,ab,kw OR (Fibroses, Oral Submucous):ti,ab,kw OR (Fibrosis, Oral Submucous):ti,ab,kw OR (Oral Submucous Fibroses):ti,ab,kw OR (Submucous Fibroses, Oral):ti,ab,kw (Word variations have been searched) 232

#2 MeSH descriptor: [Oral Submucous Fibrosis] explode all trees 81

#3 #1 or #2 or #3 241

Date Run: 31/03/2024

**Results: 241**

**Database: Embase via Ovid**

Searches:

1 ("Fibroses, Oral Submucous" or "Oral Submucous Fibrosis" or "Fibrosis, Oral Submucous" or "Oral Submucous Fibroses" or "Submucous Fibroses, Oral" or "Submucous Fibrosis, Oral").af.

2 Oral Submucous Fibrosis.mp. or Oral Submucous Fibrosis/

3 1 or 2

Date Run: 31/03/2024

**Results: 1142**
