# Supplementary figures and images for: Prevalence of oral submucous fibrosis across diverse populations: a systematic review and meta-analysis
Source: PeerJ. 2024 Nov 6;12:e18385. doi: 10.7717/peerj.18385 (PMC11549909; doi:10.7717/peerj.18385)

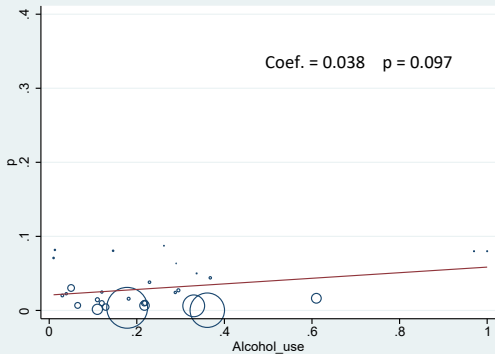

Supplement: Supplemental Information 3 — The meta-regression analysis of alcohol use, with a p > 0.001, is not considered a source of heterogeneity. [file peerj-12-18385-s003.pdf]

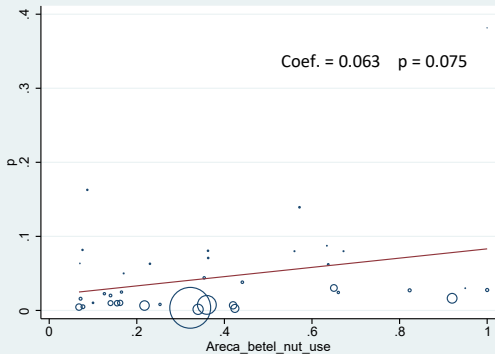

Supplement: Supplemental Information 4 — The meta-regression analysis of areca (betel) nut use, with a p > 0.001, is not considered a source of heterogeneity. [file peerj-12-18385-s004.pdf]

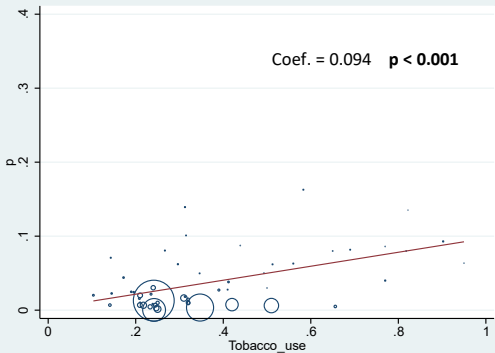

Supplement: Supplemental Information 5 — The meta-regression analysis of tobacco use, with a p < 0.001, can be considered a source of heterogeneity. [file peerj-12-18385-s005.pdf]
