# Supplementary material for: Prevalence of oral submucous fibrosis across diverse populations: a systematic review and meta-analysis
Source: PeerJ. 2024 Nov 6;12:e18385. doi: 10.7717/peerj.18385 (PMC11549909; doi:10.7717/peerj.18385)
Supplement: Supplemental Information 8 [file peerj-12-18385-s008.docx]

1. The rationale for conducting the systematic review / meta-analysis;

Oral Submucous Fibrosis (OSF) is an irreversible, chronic, and progressive precancerous oral condition, with betel nut chewing currently considered the primary etiological factor. It restricts oral function and has a relatively high rate of progression to Oral Squamous Cell Carcinoma (OSCC), severely affecting the quality of life and survival of patients. Currently, OSF cannot be cured by any effective treatment method. Prevention remains the sole viable option for clinicians to address OSF. The rationale for conducting a systematic review and meta-analysis of the prevalence of OSF across diverse populations is to synthesize existing research on the topic, providing a comprehensive and unbiased overview of knowledge, which aids in estimating the global burden of this disease. By integrating research data from different regions and populations, it offers a more thorough and accurate estimation of the epidemiological characteristics and risk factors of OSF, which is crucial for epidemiological research and public health planning. The systematic review can also assess the quality of existing studies, identify flaws and biases in study design, and provide directions for improvement in future research. Moreover, as new research emerges, regular updates of systematic reviews and meta-analyses can help in obtaining the latest epidemiological information promptly, ensuring that clinical decisions and public health policies are based on the most current scientific evidence.

1. The contribution that it makes to knowledge in light of previously published related reports, including other meta-analyses and systematic reviews；

Research on Oral Submucous Fibrosis (OSF) has predominantly been reported in Southeast Asia, particularly in India, yet its prevalence extends beyond Asia, with cases documented in Europe and North America. However, most studies reporting the prevalence of OSF are either hospital-based or rely on rely on conveniently selected research cohorts, which may not accurately reflect the true prevalence of OSF. To date, no systematic review and meta-analysis has been conducted on the existing research of OSF prevalence to derive a comprehensive estimate across populations. The primary outcome of our study is to determine a global, integrated prevalence rate for OSF.

Furthermore, the etiology of OSF is considered multifactorial and unclear. Extensive research emphasizes that betel nut chewing, tobacco use, and alcohol consumption appear to be closely associated with the development of OSF. The prevailing theory posits betel nut use as the primary risk factor for OSF, yet not all individuals addicted to betel nut chewing develop OSF. Numerous studies are attempting to elucidate the complex relationship between the two. A previous meta-analysis reported a low prevalence of OSF among betel nut chewers (5%), contradicting the strong association between betel nut chewing and OSF. Our meta-analysis, based on the percentage of betel nut, tobacco, and alcohol use in the surveyed population, found a significant increase in OSF prevalence when these risk factors are prevalent, with the trend being most pronounced in tobacco use. Although current epidemiological studies emphasize betel nut chewing as the primary risk factor for OSF, our research suggests that betel nut may not be the sole causative agent for OSF. Therefore, previously underestimated risk factors for OSF, such as tobacco use, deserve more attention, prompting the formulation of relevant public health policies and health promotion initiatives.

Additionally, our meta-analysis significantly mitigated the adverse impact of heterogeneity on the results by stratifying the data. This meta-analysis categorized the sampling population into three groups based on sampling methods, greatly reducing the heterogeneity caused by sampling methods and bringing the pooled prevalence rate of OSF closer to the true value. Moreover, subgroup analyses based on gender, geographical region, and age revealed the epidemiological characteristics of OSF. Furthermore, subgroup analyses based on the type and extent of risk factor exposure in the surveyed population provided additional perspectives for the prevention and treatment of OSF.
